# Assessment of Waste Marble Powder on the Mechanical Properties of High-Strength Concrete and Evaluation of Its Shear Strength

**DOI:** 10.3390/ma15207125

**Published:** 2022-10-13

**Authors:** Mahmoud A. El-Mandouh, Jong-Wan Hu, Ayman S. Mohamed, Ahmed S. Abd El-Maula

**Affiliations:** 1 Civil Construction Technology Department, Faculty of Technology and Education, Beni-Suef University, Beni-Suef 62511, Egypt; 2 Department of Civil and Environmental Engineering, Incheon National University, Incheon 22012, Korea; 3 Incheon Disaster Prevention Research Center, Incheon National University, Incheon 22012, Korea; 4 Civil Engineering Department, Shoubra Faculty of Engineering, Benha University, Benha 13511, Egypt

**Keywords:** waste marble powder, shear strength, reinforced concrete beams, high-strength concrete

## Abstract

Currently, the costs of building materials, especially cement, are increasing. Waste marble powder (WMP) could be used as a cement replacement material to produce environmentally friendly concrete to help preserve resources and reduce environmental pollution. The study’s goals are (1) to evaluate the effects of using marble powder in place of cement in high-strength concrete (HSC) on the material’s mechanical properties and durability characteristics. (2) The study is expanded to assess the effect of using partial WMP on the shear behavior of HSC beams under static loads. Eight half-scale simply supported reinforced beams with and without WMP have been tested. Each beam’s cross-section was 120 × 200 mm, and each beam had a total length of 1000 mm. The ratios of the used WMP were 0%, 2.5%, 5%, 7.5% by weight, and two different stirrup ratios, 0% and 0.47%, were used. When applied to HSC beams with and without WMP, the shear strength provisions of two of the most used codes, such as the locally used Egyptian Code (ECP 207) and the internationally used American Concrete Institute’s (ACI-2019), were examined. Using the ABAQUS software, the experimental results were compared to the findings of the nonlinear finite element analysis. The results established that partial replacement of cement by WMP led to increases in the concrete’s compressive and tensile strengths of about 15% and 16%, respectively. When tested specimens were exposed to acid attack, there were slight losses in weight and compressive strength (1.25% to 2.47%) for both with and without the addition of WMP. Both the concrete with and without WMP showed the same level of water absorption. Additionally, WMP led to an enhancement in the shear capacities for all beams. Increasing the WMP ratio from 0% to 2.5%, 5%, and 7.5% increased the shear capacity by about 13%, 20%, and 28%, respectively, for beams without stirrups, while for beams with stirrups, the shear capacity improved by 12%, 19%, and 25%, respectively. The enhancement in the beams’ shear capacities could be attributed to the advanced concrete matrix produced by WMP’s extremely small particle size.

## 1. Introduction

For centuries, natural decorative stones, such as marble, have been widely used around the world, particularly in developing countries. Subsequently, when these stones are prepared for use by processing, sawing, and shaping them, a huge amount of powder waste is produced, which causes a global environmental problem. Therefore, the recycling of waste marble powder (WMP) in the building sector for the manufacture of sustainable concrete as a replacement for cement has been the subject of several studies in the past decade [1,2,3,4,5,6,7,8,9,10,11,12,13,14,15,16,17,18,19,20,21,22,23]. This trend of studies has grown to minimize final construction costs, since WMP is very cheap and could be available for free, as well as to reduce global pollution. Experimental research has examined the impact of substituting WMP for cement or sand on the mechanical performance of mortar and concrete [2]. It was concluded that 10% replacement of sand by WMP gave the maximum concrete compressive strength, compared to that of the control samples with 0% WMP, at an age of 28 days. Additionally, due to WMP’s filling capability, an additional valuable influence of WMP is obvious at an early age. The impact of using WMP as a partial replacement for cement on the mechanical characteristics of concrete and the structural behavior of reinforced concrete (RC) slabs has been examined in another experimental investigation [3]. The primary factor considered was the proportion of WMP used to replace cement in concrete mixtures, which ranged from 0 to 20 percent. The outcomes displayed that WMP enhances concrete workability, compressive strength, and tensile strength. The use of 5% WMP as cement replacement causes an increase in the concrete compressive strength, tensile strength, and modulus of elasticity of 25%, 5.26%, and 12%, respectively compared to the 0% WMP concrete at age of 56 days. Additionally, 5% WMP improved the structural behavior of the slabs by increasing their stiffness and ultimate strength in comparison to the reference slabs. A similar experimental survey to explain the characteristics of concrete that used WMP to replace cement or sand has been conducted [4]. WMP replacement ratio varied from 0% to 15% by weight. It was found that at a 0.40 water/binder ratio, the use of 10% WMP as cement replacement causes an increase in the concrete compressive and tensile strengths of 9% and 14%, respectively, compared to concrete without WMP at an age of 56 days. Additionally, the use of WMP significantly affected the bond strength between steel reinforcement bars and concrete, and at a ratio of 10% WMP, the steel–concrete bond strength was extremely enhanced. Another study has been conducted to examine the properties of concrete at the fresh and hardened stages when using WMP as a cement replacement [5]. The results demonstrated that partial replacement of cement by WMP, ranging from 10% to 15%, enhanced the fresh concrete’s workability and improved the hardened concrete’s compressive, flexural, and split tensile strengths. The calcium carbonate in WMP was the reason for the improvement in concrete compressive strength. Additionally, the increase in concrete tensile strength could be attributed to the WMP filling’s impact and the development of hydration ingredients in concrete. A similar investigation has been conducted to determine how WMP substitution for cement affects the mechanical characteristics of concrete [6]. It was demonstrated that the ideal WMP replacement ratio was approximately 12%, for which the increases in the concrete compressive and split tensile strengths were 10.29% and 23.33%, respectively when compared to 0% WMP concrete. In a similar investigation [7], it was confirmed that concrete made using 15% WMP replacement by weight of cement showed an increase in the mechanical properties of hardened concrete at a low water/binder ratio of 0.35 or 0.40. Additionally, it was concluded that WMP had a superior proportion of fines but does not have enough silica and alumina. The same findings were established by another study [8], which concluded that 5% WMP replacement of cement by weight led to an increase in the concrete compressive strength of 10% at age of 28 days. In other parallel research [9,10], it was found that satisfactory results were obtained for the mechanical properties of concrete containing WMP with replacement rates not exceeding 10%. In a compatible study [13], it was established that 10% WMP replacement of cement was the optimum percentage for producing concrete; the concrete compressive strength increased by 22.84% compared to control specimens tested at 3 months. Furthermore, according to one more study [14], the replacement of 5% cement with WMP led to an improvement in the concrete compressive strength by 11.1%. The same finding was established by additional investigation [16], where it was stated that an increase in the replacement ratio of cement by WMP from 0% to 5% led to an enhancement of the compressive and flexural strengths at 28 days by 11.11% and 6.45%, respectively. The findings also demonstrate that reinforcement steel bars in concrete made with 5% WMP have less corrosion than bars in 0% concrete. During another experimental study dealing with the replacement of fine aggregate by WMP [15], it was validated that the concrete compressive strength increased by 15.15%, 22.12%, and 30.20% when the replacement ratios of fine aggregate by WMP were 10%, 20%, and 30%, respectively when compared to the 0% WMP concrete. The long-term mechanical and physical properties of concrete made by partial replacement of cement and fine aggregate by WMP were studied through experimental research [17]. It was assessed that concrete made with WMP has a higher resistance to sulphate attack and freezing and thawing than conventional concrete without WMP. The previous result was confirmed by another study [19], where it was stated that concrete made with 10% WMP showed higher durability than reference concrete made without WMP. Finally, during an extensive review paper [23], it was emphasized that the mechanical and physical properties of concrete produced by using WMP as a partial replacement of cement by weight were enhanced and that the cost of construction was decreased.

Recently, the structural applications of concrete made by replacement of cement with another cement replacement additive to help in solving environmental pollution caused by cement production have been experimentally investigated [24,25]. Mahmoud A. El-Mandouh studied the shear strength of sixteen full-scale over-reinforced concrete beams with and without nanosilica (NS) constructed from high-strength concrete (HSC) is investigated experimentally and analytically by using the ABAQUS program [25]. The finite element program ABAQUS may be successfully used to predict the shear strength of NS concrete beams. The validity of these cement replacement additives in generating reinforced concrete with improved structural behavior has been demonstrated by the results of these tests. 

The use of waste materials, especially geopolymers, as environmentally friendly materials has been the subject of experimental study [26,27]. Red mud/carbon nanotube composites were created by the chemical vapor deposition approach by breaking down hydrocarbon gas [26]. This procedure employed red mud, a leftover from the Bayer alumina manufacturing process, as a chemical agent. The results showed that red mud/carbon nanotube is an efficient adsorbent for lead ions when compared to unprocessed red mud or pure carbon nanotube. The mechanical characteristic and structural analyses of the geopolymer nanocomposite of metakaolin–red mud/carbon nanotubes were experimentally examined [27]. Metakaolin and red mud were used to create the geopolymer, in which the red mud replaced between 10 and 30 percent of the metakaolin. According to the findings, curing time enhanced the specimens’ compressive and flexural strengths. Additionally, the compressive and flexural strengths of the geopolymer specimens were decreased by the addition of red mud due to the lower reactivity of red mud and the presence of a non-reactive impurity. When adding 2% of multi-wall carbon nanotubes, the compressive and flexural strengths were enhanced by 37.05% and 36.06%, respectively, which could be attributed to the processes of crack-bridging and filling porosity and voids.

## 2. Research Objectives 

The study’s goals are (1) to evaluate the effects of using marble powder in place of cement in high-strength concrete (HSC) on the material’s mechanical and durability characteristics. (2) The study is expanded to assess the effect of using partial WMP on the shear behavior of HSC beams under static loads. For this aim, eight half-scale HSC beams with varied WMP% ratios were tested under static loading conditions. The percentage ratios of used WMP were 0%, 2.5%, 5%, and 7.5% by weight. In addition, the beams were constructed with and without stirrups to validate the contribution of WMP in concrete shear resistance when stirrups are not present. Two of the most used codes, such as the locally used Egyptian Code (ECP 207) [28] and the internationally used American Concrete Institute’s (ACI-2019) [29], were examined for shear strength requirements when applied to beams with and without WMP. Eventually, an analytical examination using the non-linear finite element computer software ABAQUS [30] has been done to compare the experimental consequences of the tested beams.

## 3. Materials and Procedures

### 3.1. Test Materials

The used cement was ordinary Portland cement with the commercial name CEM I 52.50 N, which has a specific gravity of 3.10 g/cm^3^ and an average particle size of 20.67 µm. An identical batch of cement was utilized for all the tests. Table 1 provides information about the cement’s chemical compositions. Natural river sand was utilized as the fine aggregate in this experiment. The specific gravity, water absorption, and fineness modulus of the sand were 2.45 g/cm^3^, 1.91%, and 2.23, respectively. The coarse aggregate used was 8 mm crushed limestone. Table 2 lists the physical properties of the coarse aggregates. Fresh potable water was used for mixing and curing. The used waste marble powder was attained from local marble companies, where the sawing and polishing of marble blocks produced WMP during manufacturing. Table 1 and Table 3 show the chemical composition and the physical properties of the used WMP, respectively. To achieve homogeneous dispersion of the concrete materials and to validate workability in fresh conditions, a superplasticizer (SP) was utilized. To guarantee the necessary workability, a superplasticizer was required. This superplasticizer, which complies with ASTM C494 type F [31], is made of synthetic resins and was added at a dose of 1.5% by weight of cement to increase the workability of concrete. The percentage ratios of used WMP were 0%, 2.5%, 5%, and 7.5% by weight. Table 4 illustrated the used concrete mixes’ designs. For the mix proportion mentioned above, 75 mm of the slump was obtained.

### 3.2. Test Specimens

Three concrete cylinders (ϕ150 × 300 mm) and three concrete cubes (150 × 150 × 150 mm) were taken for each percentage of the WMP mix to determine the average cylinder compressive strength (*f_c_*^′^) and the average cube compressive strength (*f*_cu_), respectively. The compressive strength of the concrete cylinder or cube is measured as a continuous load applied to the specimen till failure. To determine the average concrete tensile strength (*f_t_*), three more concrete cylinders were taken for each ratio of the WMP. The tensile test is an indirect method for assessing the tensile strength of concrete. In this test, the concrete cylinder is set out horizontally, and a force is applied radially on its surface, causing a vertical fracture to develop along the cylinder’s diameter. Forty-five cube specimens for each mixture (100 × 100 × 100 mm) were cast to determine durability characteristics, such as acid resistance and water absorption. 

### 3.3. Preparation of Test Specimens

The drum was filled with dry-mixed cement, sand, marble powder, and coarse aggregate. Water was then gently added and properly blended. The specimens were cast in steel molds and compacted using a needle vibrator. After casting for 24 h, each specimen was removed from the mold, cured for 90 days, and then tested. 

## 4. Specimens Results

### 4.1. Concrete Strengths 

**Figure 1:**
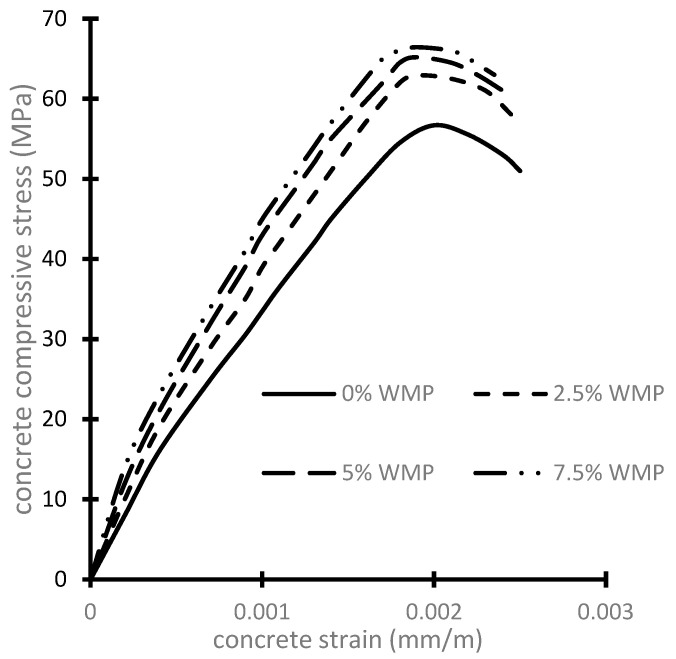
Concrete stress–strain relations.

**Figure 2:**
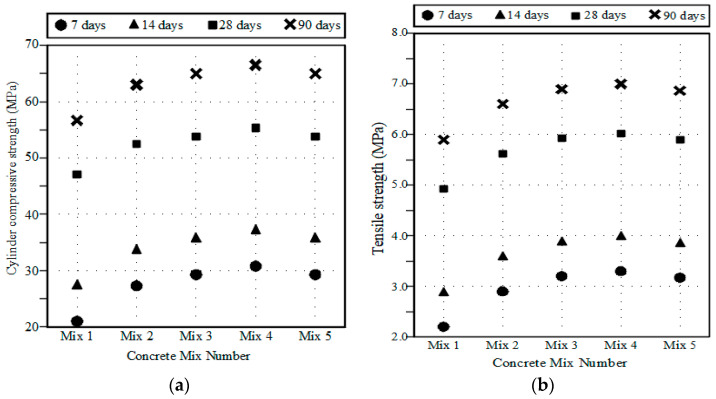
Test specimens’ strengths. (**a**) Cylinder compressive strength; (**b**) Tensile strength.

The average concrete compressive and tensile strengths for each WMP ratio are given in Table 5. The average stress–strain curves for HSC with and without WMP are shown in Figure 1. The stress–strain curve’s ascending branch was significantly affected by the increase in the WMP ratio. The concrete strengths of all the test specimens are presented in Figure 2. It was found that increasing the WMP ratio from 0% to 2.5%, 5%, and 7.5% led to an increase in the concrete’s compressive strength by about 11%, 15%, and 17% respectively. Additionally, the concrete’s tensile strength was increased by 13%, 17%, and 19% at WMP ratios of 2.5%, 5%, and 7.5% respectively in comparison with the reference no-WMP concrete. The increase in concrete compressive strength by using WMP as cement replacement was emphasized in previous experimental studies [2,3,4,5,6,7,9,10,14]. Additionally, the enhancement of the concrete’s tensile strength was highlighted in several earlier experimental investigations [4,5,6,8]. The enhancement of concrete compressive strength could be attributed to the presence of calcium carbonate in WMP, which results in the concrete matrix’s densification and pore reduction. Additionally, the rise in concrete tensile strength might be explained by the WMP filling’s impact and the development of hydration ingredients in concrete. Moreover, the extremely high fineness of WMP makes it particularly useful for verifying the exceptional cohesiveness of concrete. On the other hand, the experimental results showed that the concretes’ strengths were decreased at WMP ratios of 10% in comparison to specimens that have 7.50% WMP. This could be attributed to the fact that when the ratio of cement replacement by WMP exceeds 7.50%, the less reactive calcite has a weakening impact on the further reactive cement; the amount of cement paste is significantly decreased, causing decreased compressive strength. In addition, the experimental results showed that the tensile concrete strengths were decreased at WMP ratios of 10% in comparison to specimens that have 7.50% WMP. According to these results, it could be established that the optimum ratio of the used WMP is 7.50% because the concrete compressive and tensile strengths were decreased for a higher ratio. The same findings established that the maximum dosage of WMP should exceed 10% by weight of cement [9,10,13].

### 4.2. Acid Attack

**Figure 3:**
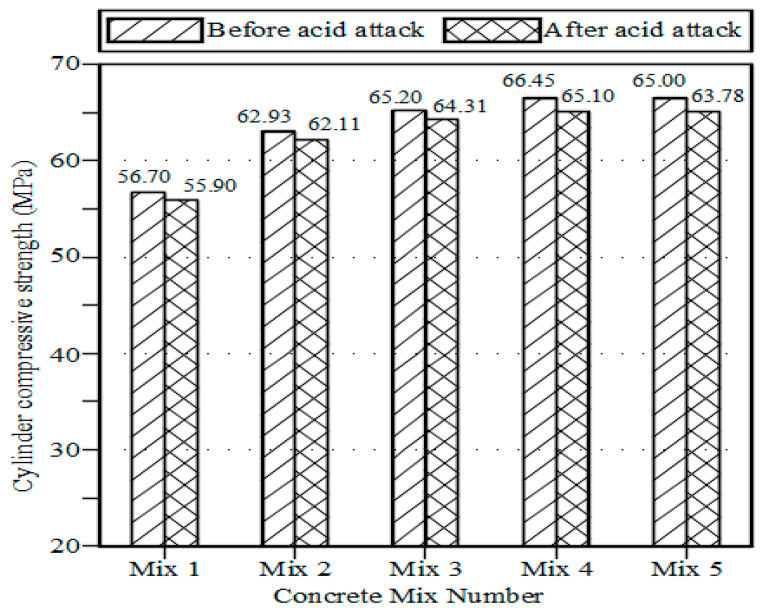
Acid attack test results.

**Figure 4:**
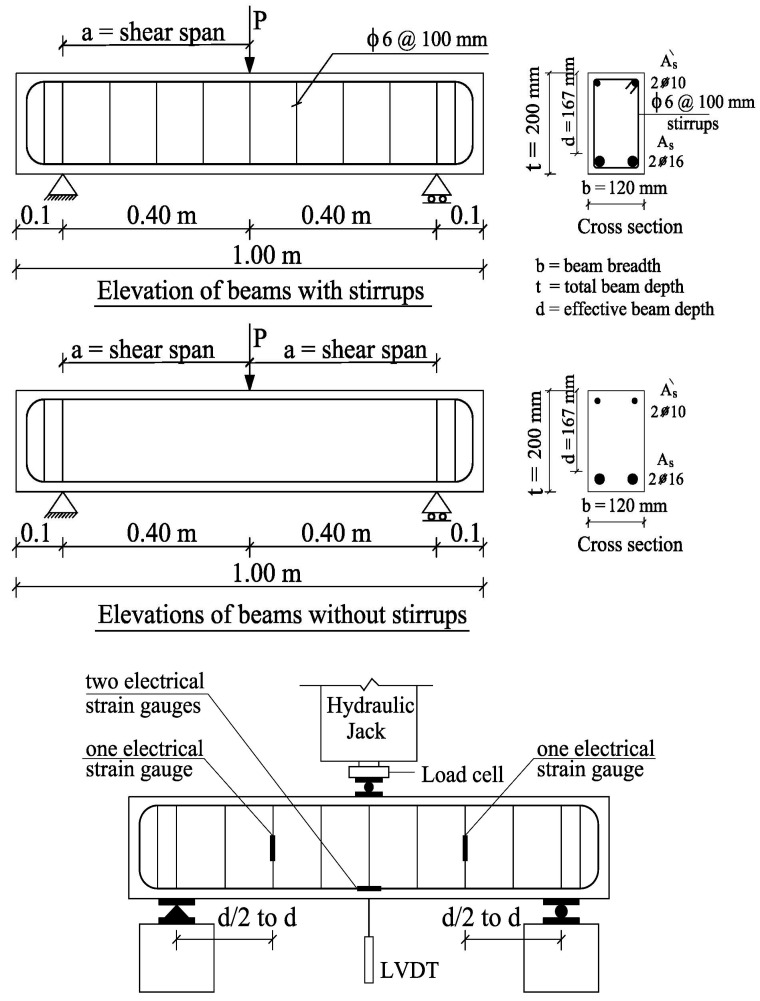
Details of the tested beams and test setup.

The acid solution was created by combining distilled water with 3% sulfuric acid (H_2_SO_4_) and 2% hydrochloric acid (HCl). The specimens were air dried, chilled at ambient temperature, weighed using an electronic balance, and then submerged into an acid bath after the 28-day curing period. Before immersion, the initial weights of each specimen were noted. The weight losses and compressive strengths of conventional and marble powder concrete specimens were measured after immersion for 15 days in acid, and the results are shown in Figure 3. None of the test specimens’ measured weights significantly decreased. However, as seen in Figure 4, there were slight decreases in compressive strengths.

### 4.3. Water Absorption Test

Table 6 illustrates the water absorption percentages for ordinary and marble-powdered concrete cubes. In terms of water absorption, there was no discernible difference between conventional and marble-powdered concrete cubes.

## 5. Experimental Program

The study is expanded to assess the effect of using partial WMP on the shear behavior of HSC beams under static loads.

### 5.1. Beams Description

Experimental testing was performed on eight simply supported waste marble powder high-strength reinforced concrete beams. To divide the beam into two identical shear spans, all beams were tested with a single concentrated load at the mid-span point. As mentioned above, the concrete strengths were decreased at WMP ratios of 10% in comparison to specimens that have 7.50% WMP. Thus, the percentages ratios of used WMP were 0%, 2.5%, 5%, and 7.5% by weight with the concrete mix are illustrated in Table 4. Table 7 and Figure 4 provide the details of the tested beams’ concrete size and reinforcing. The applied shear-span-to-effective depth (a/d) ratio was 2.4, while the two vertical stirrups ratios (*ρ*_v_) were 0% and 0.47%. Vertical stirrups were constructed using 100 mm-spaced, 6 mm diameter bars with 240 MPa yield stress. According to ACI Code provisions (ACI-2019) [29] for the design of reinforced concrete sections, all beams were over-reinforced to prevent premature flexure failure. Two 16 mm diameter bars with a 410 MPa design yield stress were used as the main bottom longitudinal reinforcement, while two 10 mm diameter bars with the same yield stress were used as the top longitudinal reinforcement. The top and bottom longitudinal bars were bent at the beam edges with legs equal to the depth of the beam. All beams were tested at an age of 90 days. 

### 5.2. Test Setup 

Each specimen was examined using a standard universal machine. The loads were measured using a load cell with a 300 kN capacity and 0.1 kN precision. Figure 4 shows the experimental test setup as well as the linear variable differential transformer (LVDT) and electrical strain gauge locations. On the right and left ends of the examined beams, roller and hinged supports were employed, respectively. Measurements were made for the stirrups’ strain at the center of the shear span, the steel’s mid-span strain, and the beam’s central deflection.

## 6. Experimental Results and Discussion

### 6.1. Beams Crack Patterns 

**Figure 5:**
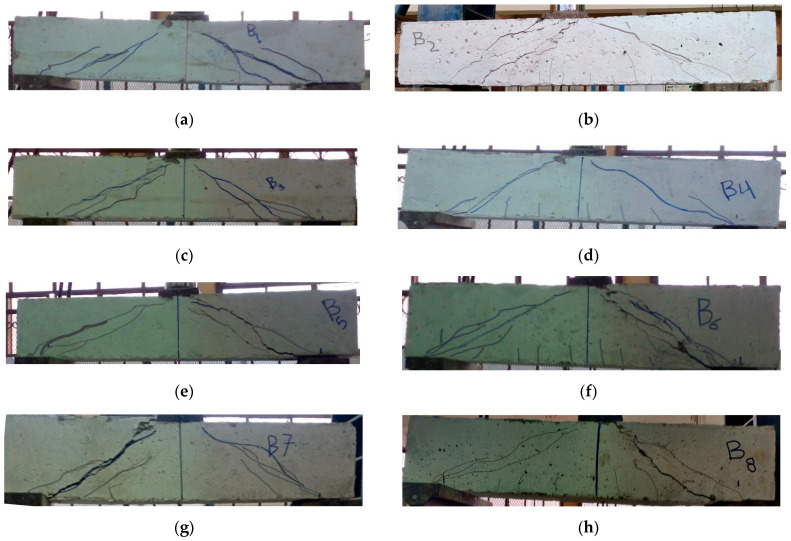
The crack patterns of the tested beams. (**a**) Crack pattern of beam B1; (**b**) Crack pattern of beam B2; (**c**) Crack pattern of beam B3; (**d**) Crack pattern of beam B4; (**e**) Crack pattern of beam B5; (**f**) Crack pattern of beam B6; (**g**) Crack pattern of beam B7; (**h**) Crack pattern of beam B8.

The failure cracking patterns of the tested beams are illustrated in Figure 5; additionally, the cracking load (P_cr_) and the ultimate failure load (P_u_) for each beam are given in Table 7. As all beams were over-reinforced to avoid premature bending failure, all test beams under shear failed before stressing the main bottom longitudinal reinforcement. For beams without stirrups, the shear failure was brittle and rapidly occurred. For all specimens, the initial crack was almost vertical and began in the central zone of the beam then further approximately vertical cracks occurred near the beam mid-span. As the load increased, an inclined crack originated in the shear span zone between the point of load and the point of the support. At failure, this diagonal crack expanded suddenly, causing the beams to fail in shear. It was feasible to determine that an increase in WMP ratio resulted in an almost decreased crack number, increased crack spacing, and decreased crack width. These results were more noticeable for beams without stirrups, for which shear resistance is solely determined by the concrete. Additionally, compared to specimens without stirrups, the specimens with stirrups had more cracks with reduced spacing, but the widths of cracks were smaller. In addition, the output data established that raising the WMP ratio delayed P_cr_ with respect to P_u_. It should be emphasized that the formation of diagonal cracks inside the beam’s web, which becomes unstable and breaks, is the primary reason for the shear failure of the reinforced concrete beams. Therefore, the compressive and tensile strengths of the concrete utilized have a major impact on the shear failure mechanism of the beam. Consequently, as the WMP ratio increased, the concrete compressive and tensile strengths increased, and the beam’s shear capacity increased. 

### 6.2. Loads vs. Vertical Deflections 

**Figure 6:**
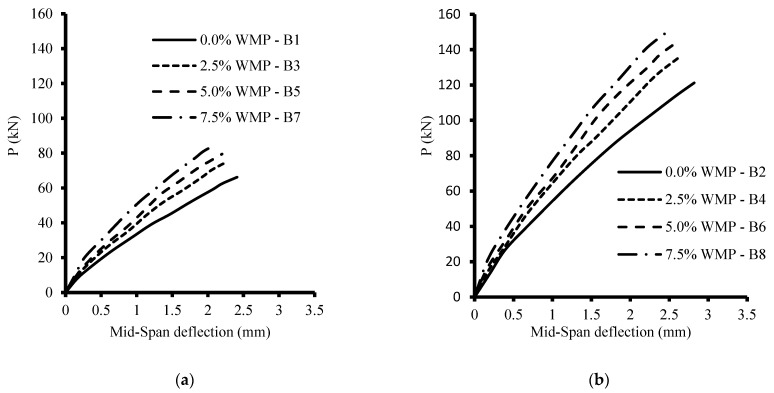
The load-deflection relations of the tested beams. (**a**) without stirrups, ***ρ***_v_ = 0%; (**b**) with stirrups, ***ρ***_v_ = 0.47%.

The load-deflection relations were recorded for all tested specimens and are displayed in Figure 6. Additionally, the maximum mid-span deflection (Δ_u_) corresponding to the ultimate failure load (P_u_) is presented in Table 7. In general, increasing the WMP ratio increased the load capacities of all levels, but it also decreased the vertical mid-span deflections for the same load level. It was detected that increasing the WMP ratio from 0% to 2.5%, 5%, and 7.5% increased P_u_ by about 13%, 20%, and 28%, respectively, for beams without stirrups, while for beams with stirrups, the ultimate load improved by 12%, 19%, and 25%, respectively. It could be confirmed that the use of WMP increased the shear resistance because the WMP is vital to increasing the compressive and tensile strengths of concrete, which enhance shear resistance.

### 6.3. Stirrups Strains 

**Figure 7:**
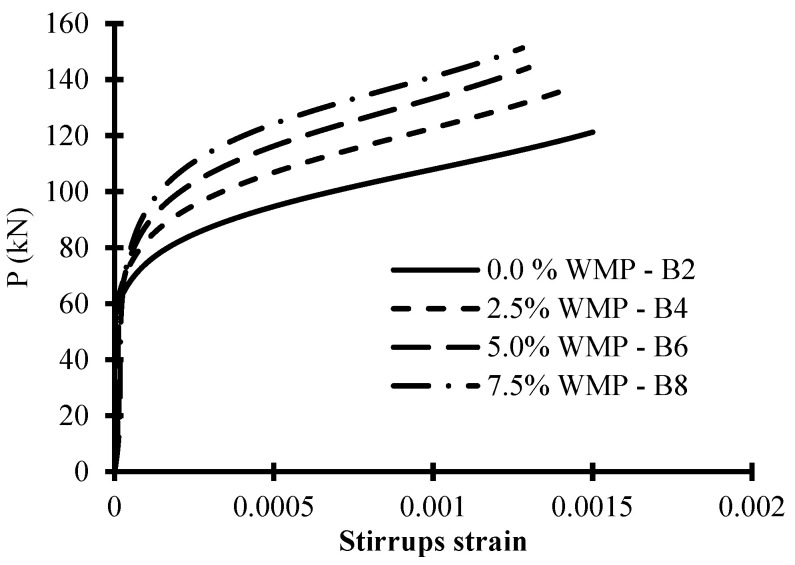
The load–stirrup strain relations of the tested beams with stirrups.

To show the influence of adding WMP on the stirrup strains, the curves of load versus tensile stirrups strain are shown in Figure 7. The strain in the stirrups was measured in their vertical legs when they passed the failure plane, which is almost in the middle of the shear span. Figure 7 shows that when the WMP ratio increased, the load-carrying capacity increased and stirrup strain decreased at various load levels. For stirrup-equipped specimens, raising the WMP ratio from 0% to 2.5%, 5.0%, and 7.5% resulted in a decrease in the stirrups strain of about 16%, 22%, and 27%, respectively, at the failure load level. This might be attributed to the fact that boosting WMP strengthened the concrete’s tensile properties, which reduced tension on the stirrups.

## 7. Code Provisions

### 7.1. ACI 318-19 

According to the ACI code [29], the critical section of shear is at “d” taken from the column face. The total shear force capacity (V_u_) in a concrete beam reinforced with stirrups, which is resisted by both the concrete (V_c_) and the stirrups (V_s_), is given by the equations below:

V_u_ = V_c_ + V_s_ (1)

V_c_ = 0.17 λ (*f_c_*′)^0.5^ b d (2)

V_s_ = A_v_
*f_ys_* d/s (3)

where λ is taken as “1.0” for normal-weight concrete; *f_c_*′ is the concrete compressive strength of the cylinder in MPa; b is the beam width in mm; d is the effective beam depth in mm; s is the spacing between vertical stirrups in mm; A_v_ represents the shear reinforcement area (mm^2^); and *f_ys_* is the stirrups’ yield stress in MPa.

### 7.2. ECP 207 

According to ECP Code [28], the critical section of shear is at “d/2” taken from the column face. The total shear force capacity (V_u_) is computed as per ACI 318-19, except for the concrete shear force (V_c_), which has the following calculation:

V_c_ = 0.16 (*f_cu_*/γ_c_)^0.5^ b d (4)

where *f_cu_* is the concrete compressive strength of the cube in MPa, which is about 1.25 times *f_c_*′, and γ_c_ is a strength reduction factor for concrete and equal to 1.50.

## 8. Test Results and Code Predictions Comparison

The previous section, “Code Provisions”, can be considered an introduction to the current section. As shown in Table 8, the tested specimens’ shear failure experimental forces (V_u exp._) were compared with those determined from the ACI 318-19 (V_u ACI_) and ECP 207 (V_u ECP_) codes. As depicted in Table 8, compared with beams with a low percentage of WMP, the studied codes are safer in expecting ultimate shear capacity for beams with a high WMP ratio. Additionally, when compared to beams without stirrups, the studied codes are slightly safer for calculating WMP concrete contribution for shear. The ratios between the experimental shear failure force and that determined from ACI 318-19 and ECP 207 had average values of 1.40 and 1.59, respectively, which indicates that the ECP Code is more conservative when designing shear compared to the ACI Code.

## 9. Finite Element Modeling 

The ABAQUS program was used to carry out finite element simulations of the tested reinforced concrete beams. Nonlinearity in the materials was considered and described as follows.

### 9.1. Material Nonlinearity

The concrete damage plasticity (CDP) model is utilized in this study to explain the behavior of the concrete beams. Figure 1 illustrates the nonlinear stress–strain relationship of concrete under compression. The concrete damaged plasticity material model’s stiffness degradation includes coefficients for compression (d_c_) and tension (d_t_). When the concrete material reaches its maximal stress, they depict the progression of the stresses in concrete. Table 9 provides a list of the CDP model’s input parameters that were employed in the current study. The tensile stress σ_t_ was calculated according to Eurocode 2 [32] as:

σ_t_ = *f_t_* [*ε_t_*/*ε*]^0.4^ (5)

where *ε_t_* is the tensile strain in the concrete at the peak stress *f_t_*, ε is the tensile strain in the concrete, and *f_t_* is the concrete tensile strength, which is taken from experimental results. For the steel longitudinal bars in the finite element model, an elastic completely plastic model with equal behavior in tension and compression was adopted. ABAQUS software defined the Young modulus (E_s_) and Poisson’s ratio (*v*) as equal to 2 × 10^5^ MPa and 0.30, respectively, with yield stress *f_y_* for longitudinal and transverse bars, as illustrated in the experimental program.

### 9.2. Types of Elements

**Figure 8:**
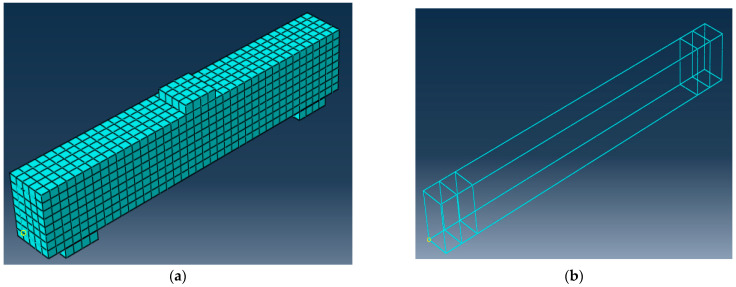
Meshes of some tested beams. (**a**) Concrete volume meshes; (**b**) Reinforcement meshes.

The solid elements (C3D8R) from the ABAQUS library are used to model the concrete beam. They are reduced integration, eight-node elements with hourglass control, and each node has three degrees of freedom for translation as well as a truss element (T3D2) to simulate reinforcing bars. It consists of two nodes with three degrees of freedom each (translations in X, Y, and Z directions). In this study, embedded technology was used to create frictional contact between the steel and concrete in ABAQUS [33]. To choose an appropriate mesh with acceptable accuracy in terms of ultimate load and ultimate deflection, three models with various fine, medium, and coarse mesh sizes of 15, 25, and 35 mm, respectively were examined for all elements. The concrete medium volume meshes, and bar reinforcement meshes for some tested beams are illustrated in Figure 8.

### 9.3. Loading and Boundaries Conditions

**Figure 9:**
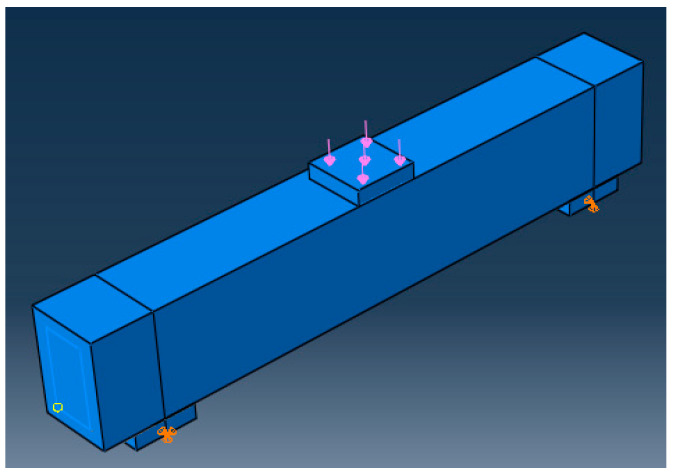
Loading and boundary conditions.

The load was applied as a one-point load, and the supports of tested beams are roller support (U_1_ = U_2_ = 0.0) and hinged support (U_1_ = U_2_ = U_3_), as illustrated in Figure 9, where U_1_, U_2_, and U_3_ are translation in X, Y, and Z directions, respectively, to mimic the experimental test setup.

## 10. Comparison of the Experimental and Finite Element Findings

**Figure 10:**
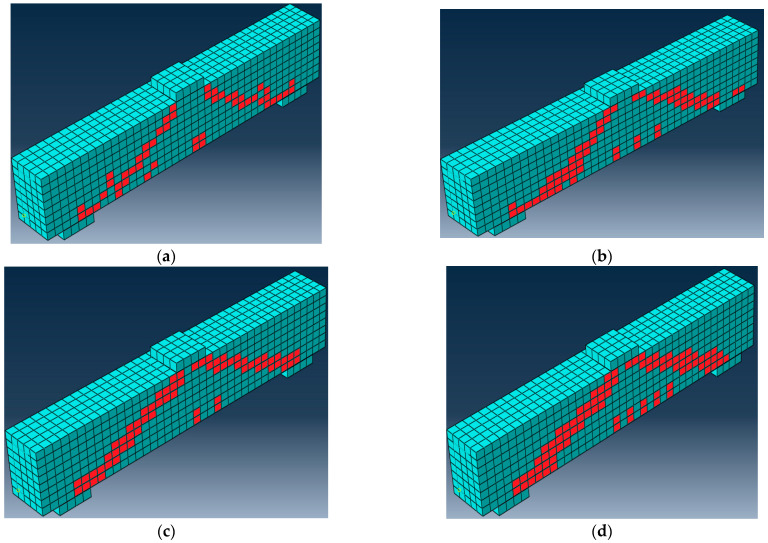
FE final cracks of some specimens. (**a**) Specimen B1; (**b**) Specimen B2; (**c**) Specimen B5; (**d**) Specimen B6.

**Figure 11:**
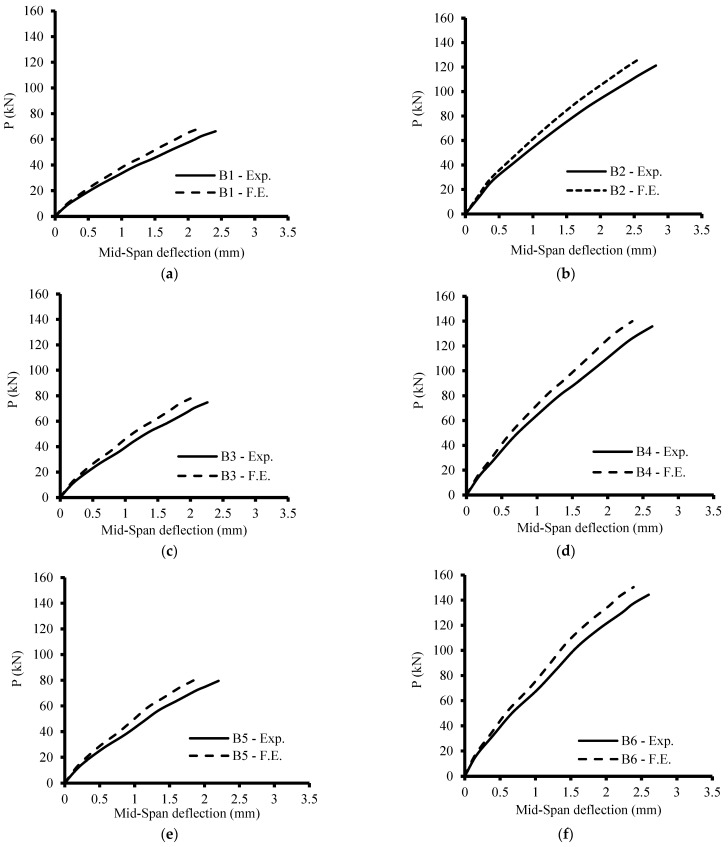

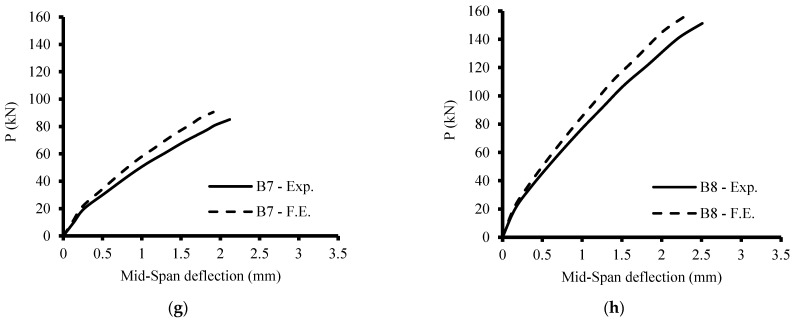
Load deflection curves of the tested specimens (numerical and experimental results). (**a**) Specimen B1; (**b**) Specimen B2; (**c**) Specimen B3; (**d**) Specimen B4; (**e**) Specimen B5; (**f**) Specimen B6; (**g**) Specimen B7; (**h**) Specimen B8.

It has been found that the medium mesh gives the best results in the ultimate load and deflection values, as shown in Table 10. Additionally, it has an acceptable computational time compared to the other meshes. The range of 0.94 to 0.97 was the FE estimate for the experimental ultimate load ratio, as illustrated in Table 10. Related to the experimental results, the load-deflection curves from numerical simulations behave more stiffly after the first crack, which is likely caused by a sizable scatter in the tensile strength of the concrete. Figure 10 shows the final finite element cracks of some of the examined beams. Figure 11 displays the numerical model’s load–deflection relations and the related experimental findings. The average ratio of the experimental to the FE ultimate load was 0.96, while the average ratio of the experimental to the FE mid-span displacement was 1.11. The findings of theoretical finite elements were somewhat bigger than the values from experiments. These variations might be a result of the experiment’s human measurement errors. Additionally, a stiffer finite element model could result from assuming a perfect bond between the concrete surface and reinforcement bars. This indicates that the experimental findings and FE simulations were in good agreement.

## 11. Conclusions 

The results of the experimental investigations and analytical analyses of the shear behavior of WMP reinforced concrete beams can be used to draw the following conclusions:

- Increasing the WMP ratio from 0% to 2.5%, 5%, and 7.5% led to an increase in the concrete compressive strength by about 11%, 15%, and 17%, respectively. In addition, the concrete tensile strength was increased by 13%, 17%, and 19% at the same WMP ratios.
- The increase in the WMP ratio resulted in a roughly decreased crack number, increased crack spacing, and decreased crack width.
- Increasing the WMP ratio from 0% to 2.5%, 5%, and 7.5% increased P_u_ by about 13%, 20%, and 28%, respectively, for beams without stirrups, while for beams with stirrups, the ultimate load improved by 12%, 19%, and 25%, respectively.
- When tested specimens were exposed to acid attack, there were slight losses in weight and compressive strength (1.25% to 2.47%) for both with and without the addition of WMP.
- Both the concrete with and without WMP showed the same level of water absorption.
- For stirrup-equipped specimens, raising the WMP ratio from 0% to 2.5%, 5.0%, and 7.5% resulted in a decrease in the stirrup strain of about 16%, 22%, and 27%, respectively.
- The average values of the ratios between the experimentally measured shear failure force and those derived from ACI 318-19 and ECP 207 were 1.40 and 1.59, respectively, indicating that the ECP Code is more conservative when designing shear than the ACI Code.
- The average ratio of the experimental to the FE ultimate load was 0.96, while the average ratio of the experimental to the FE mid-span displacement was 1.11. This indicates that the experimental findings and FE simulations were in good agreement.

## Figures and Tables

**Table 1:** Chemical compositions of cement and WMP.

Chemical Composition	SiO_2_	Al_2_O_3_	Fe_2_O_3_	CaO	SO_3_	K_2_O	Na_2_O	MgO	CL
% Cement	19.39	4.13	4.70	55.66	3.9	0.28	0.31	1.70	-
% WMP	21.44	5.50	3.65	62.24	2.99	0.60	0.90	2.65	0.03

**Table 2:** Physical properties of coarse aggregates.

Characteristic	Specific gravity	Water absorption	Fineness modulus	Crushing strength	Impact strength
	19.39	4.13	4.70	55.66	3.9

**Table 3:** Physical properties of WMP.

Density (g/cm^3^)	Specific Gravity	Fineness (m^2^/kg)	Color
2.78	2.67	350	Light gray

**Table 4:** Concrete mixes’ designs.

Mix No.	WMP %	Cement kg/m^3^	WMP kg/m^3^	SP kg/m^3^	Fine Aggregate (Sand) kg/m^3^	Coarse Aggregate (Crushed Limestone) kg/m^3^	Water kg/m^3^
1	0.0	500	0	5.00	681	1065	179
2	2.5	487.5	12.5	4.88	681	1065	179
3	5.0	475	25	4.75	681	1065	179
4	7.5	462.5	37.5	4.63	681	1065	179
5	10	450	50	4.50	681	1065	179

**Table 5:** Concrete mixes’ design results.

Mix No.	WMP %	*f_c_*′(MPa)	*f_cu_* (MPa)	*f_t_* (MPa)
1	0.0	56.70	70.81	5.91
2	2.5	62.93	78.65	6.68
3	5.0	65.20	81.48	6.90
4	7.5	66.45	83.11	7.03
5	10	65.00	81.25	6.87

**Table 6:** Results of water absorption tests.

Mix No.	WMP %	Water Absorption (%)
1	0.0	0.75
2	2.5	1.12
3	5.0	1.35
4	7.5	1.56
5	10	1.67

**Table 7:** Description of tested specimens.

Serial	Beam Name	WMP %	b mm	d mm	Stirrups ϕ Bar Diameter mm @ Spacing (mm)	*ρ_v_* %	P_cr_ kN	P_u_ kN	Δ_u_ mm	P_cr_/P_u_
1	B1	0.0	120	167	0	0.00	46.36	66.25	2.41	0.70
2	B2				ϕ 6 @ 100	0.47	77.60	121.25	2.82	0.64
3	B3	2.5	120	167	0	0.00	53.84	74.86	2.26	0.72
4	B4				ϕ 6 @ 100	0.47	86.90	135.80	2.63	0.64
5	B5	5.0	120	167	0	0.00	58.14	79.51	2.20	0.73
6	B6				ϕ 6 @ 100	0.47	94.70	144.33	2.60	0.66
7	B7	7.5	120	167	0	0.00	63.10	85.11	2.12	0.74
8	B8				ϕ 6 @ 100	0.47	102.34	151.32	2.51	0.68

**Table 8:** Comparison of experimental results with the examined codes.

Beam	V_u exp._ = (P_u_/2) (kN)	V_u ACI_ (kN)	V_u ECP_ (kN)	V_u exp._/V_u ACI_	V_u exp._/V_u ECP_
B1	33.13	25.65	22.04	1.29	1.50
B2	60.63	48.31	44.69	1.26	1.36
B3	37.43	27.03	23.22	1.38	1.61
B4	67.90	49.68	45.87	1.37	1.48
B5	39.76	27.51	23.63	1.45	1.68
B6	72.17	50.16	46.29	1.44	1.56
B7	42.56	27.77	23.86	1.53	1.78
B8	75.66	50.42	46.51	1.50	1.63
Average value				1.40	1.59

**Table 9:** CDP model parameters under compound stress.

Parameter	Value
Angle of dilatation	37°
Eccentricity	0.1
The ratio of initial equibiaxial compressive yield stress to initial uniaxial compressive yield stress (*f_bo_*/*f_co_*)	1.16
The ratio of the second stress invariant on the tensile meridian (K)	0.667
Viscosity parameter	0.0001

**Table 10:** Comparison of the experimental and finite element findings.

Specimen	Failure Load P_u_, kN				Maximum Mid-Span Displacement (mm)			
	Finite Element			EXP./Finite Element (Medium)	Finite Element			EXP./Finite Element (Medium)
	Fine	Medium	Coarse		Fine	Medium	Coarse	
B1	67.57	64.26	62.92	0.97	2.92	2.69	2.59	1.12
B2	122.32	116.40	114.02	0.96	3.36	3.10	2.99	1.10
B3	75.55	71.86	70.37	0.96	2.78	2.55	2.45	1.13
B4	138.41	131.72	129.04	0.97	3.19	2.94	2.83	1.12
B5	80.25	76.33	74.75	0.96	2.70	2.48	2.38	1.13
B6	145.58	138.55	135.73	0.96	3.07	2.83	2.72	1.09
B7	84.10	80.00	78.35	0.94	2.57	2.35	2.25	1.11
B8	154.22	146.78	143.79	0.97	3.00	2.76	2.65	1.10
		Average		0.96		Average		1.11

## Data Availability

All the data supporting reported results can be found in the manuscript.

## Nomenclature

A_s_	the area of the main bottom steel reinforcement bars in tension
A_s_^′^	the area of secondary top steel reinforcement bars in compression
A_v_	the shear reinforcement area
a	shear span
b	breadth of the beam cross section
d	effective depth of the beam cross section
d_c_	concrete damaged plasticity material model’s stiffness degradation coefficients for compression
d_t_	concrete damaged plasticity material model’s stiffness degradation coefficients for tension
E_s_	Young steel modulus
*f_cu_*	concrete cube compressive strength
*f_c_* ^′^	concrete cylinder compressive strength
*f_t_*	concrete tensile strength
*f_y_*	yield stress of the main reinforcing bars
*f_ys_*	the stirrups yield stress
*f_bo_*/*f_co_*	the ratio of initial equibiaxial compressive yield stress to initial uniaxial compressive yield stress
K	The ratio of the second stress invariant on the tensile meridian
P	applied load
P_cr_	cracking load
P_u_	ultimate failure load
s	the spacing between vertical stirrups
t	total depth of the beam cross section
V_c_	concrete shear force capacity
V_s_	stirrups shear force capacity
V_u_	total beam shear force capacity
V_u ACI_	beam shear capacity according to ACI 318-19
V_u ECP_	beam shear capacity according to ECP 207
V_u exp._	beam experimental shear capacity
*ε*	concrete tensile strain
*ε_t_*	concrete tensile strain at the peak stress *f_t_*
Δ_u_	maximum mid-span deflection
*ρ*_v_	vertical stirrups ratio
λ	Factor taken as 1.0 for normal-weight concrete according to ACI 318-19
γ_c_	strength reduction factor for concrete and equal to 1.50 according to ECP 207
σ_t_	concrete tensile stress
*v*	Poisson’s ratio
